# Flotation Properties and Surface Interactions of a Multicomponent Xanthate Collector Based on Fusel Oil in the Flotation of Sulfide Minerals

**DOI:** 10.3390/ma19214634

**Published:** 2026-10-31

**Authors:** Nazira Samenova, Nessipbay Tussupbayev, Dulatbek Turysbekov, Bakyt Daulet

**Affiliations:** Institute of Metallurgy and Ore Beneficiation JSC, Satbayev University, Almaty 050010, Kazakhstan; nazira.orakkyzy@gmail.com (N.S.); dula80@mail.ru (D.T.); b.d.e.metal@mail.ru (B.D.)

**Keywords:** multicomponent xanthate, fusel oil, flotation, sulfide minerals, flotation kinetics, surface interaction

## Abstract

Developing effective collectors for the flotation separation of sulfide minerals is of interest for enhancing the recovery of valuable components from mineral raw materials. This study investigates the flotation properties and surface interactions of a multicomponent xanthate (MX) collector derived from fusel oil. The composition of the starting fusel oil was determined via gas chromatography–mass spectrometry (GC-MS), while the resulting MX was characterized using ^1^H and ^13^C nuclear magnetic resonance (NMR) and Fourier transform infrared (FTIR) spectroscopy. The flotation activity of MX was evaluated using chalcopyrite, galena, sphalerite, and pyrite across three size fractions (−0.100 + 0.074, −0.074 + 0.040, and −0.040 + 0 mm) at collector concentrations of 2.5, 5.0, and 7.5 mg/L. Additionally, zeta-potential analysis, FTIR, and X-ray photoelectron spectroscopy (XPS) were employed to assess the interaction between the collector and the mineral surface. It was found that the flotation efficiency of MX depends on the mineral type, particle size, and collector concentration. The highest chalcopyrite recovery (81.9%) was achieved with the −0.074 + 0.040 mm fraction at an MX concentration of 7.5 mg/L after 3 min of flotation. Maximum final recovery values for galena, sphalerite, and pyrite were 73.9%, 51.4%, and 23.9%, respectively. The results demonstrate that the multicomponent composition of MX provides mineral-specific flotation performance, making it a promising candidate for further research in the beneficiation of sulfide mineral ores.

## 1. Introduction

The efficient beneficiation of sulfide minerals, particularly in the fine and ultrafine size ranges, remains one of the most challenging tasks in modern mineral processing technology [1]. As high-grade, easily beneficiated ores are gradually depleted, low-grade, refractory, and finely disseminated ores in which valuable minerals are intimately associated with gangue components are increasingly being processed [2]. During the flotation of such systems, process efficiency declines due to the low probability of collisions between fine particles and air bubbles, insufficient attachment strength, and the significant influence of surface and hydrodynamic factors [3,4]. Consequently, the development of new, highly efficient collectors capable of ensuring selective mineral surface hydrophobization under complex conditions is of considerable scientific and practical interest [5].

Among the collectors used in sulfide mineral flotation, xanthates hold a special place due to their high activity, capacity for chemisorption, and effectiveness across a wide range of sulfide minerals [6,7,8]. Alkali metal alkyl xanthates are the most common, particularly potassium amyl xanthate, which is widely used in the flotation of copper, lead, zinc, and other sulfide ores [9]. However, the use of traditional individual xanthates entails several limitations. These include insufficient selectivity in complex mineral systems, a dependence of efficiency on pH and the redox potential of the medium, and sensitivity to the composition of the mineral surface [10,11]. Furthermore, the use of xanthates synthesized from individual alcohols does not always allow for sufficient flexibility in adjusting the reagent’s hydrophobic and adsorption properties [12].

In recent years, particular attention has been paid to developing next-generation flotation reagents based on readily available secondary raw materials and industrial waste, aligning with modern principles of resource conservation, the circular economy, and green chemistry [13,14,15]. Utilizing industrial byproducts as feedstock for the synthesis of flotation reagents addresses two objectives simultaneously: reducing the environmental burden associated with waste accumulation and developing more cost-effective reagents with specific functional properties [16]. One promising type of such feedstock is fusel oil, a byproduct of alcohol production generated during the rectification of ethyl alcohol [17].

Fusel oil is a complex multicomponent mixture of higher aliphatic alcohols and associated organic compounds; its composition typically includes isoamyl alcohol, isobutanol, propanol, and other components [18,19]. Due to the presence of hydrocarbon radicals and hydroxyl groups, these compounds can be viewed as promising precursors for synthesizing surfactants and flotation reagents. Unlike the individual alcohols used to produce standard xanthates, fusel oil as a multicomponent feedstock enables the synthesis of composite xanthates containing a mixture of various alkyl fragments. Such structural diversity can help broaden the range of hydrophobic interactions, enhance adsorption onto mineral surfaces, and improve reagent adaptability when processing complex mineral ores [20].

This approach is particularly relevant for the beneficiation of finely dispersed sulfide minerals, where flotation efficiency is largely determined not only by the chemical nature of the collector but also by its interfacial activity, sorption characteristics, and ability to modify the electrical and wetting properties of the mineral surface [21,22,23]. In this context, it is promising to not only synthesize a new composite xanthate but also conduct a comprehensive study of its colloidal-chemical characteristics, including its effect on zeta potential, as well as the mechanism of interaction with mineral surfaces as revealed by spectroscopic methods [24,25].

Despite significant interest in using organic waste as a raw material for producing flotation reagents, the synthesis and application of composite xanthates derived from fusel oil have not been sufficiently investigated [26]. Available literature focuses heavily on traditional xanthates and other industrial collectors, whereas data on the properties of mixed xanthate systems derived from alcohol production waste remain limited [27]. The relationships between the reagent’s composition, its adsorption behavior, and its flotation activity regarding sulfide minerals are not yet fully understood [28,29,30]. This hinders the scientific justification for their use and impedes the targeted development of more effective collectors.

Accordingly, the aim of this study is to synthesize a composite xanthate collector based on fusel oil, a waste product from the Talgar Distillery, and to investigate its colloidal-chemical and flotation properties. It is hypothesized that using a multicomponent alcohol solution will yield a reagent with a broader spectrum of hydrophobicity and superior adsorption characteristics compared to standard single-component xanthates. The study evaluates the new collector’s efficacy in sulfide mineral flotation and characterizes its interaction with mineral surfaces using techniques such as IR spectroscopy, X-ray photoelectron spectroscopy (XPS), and zeta-potential measurement.

The scientific novelty of this work lies in the development of a composite xanthate reagent derived from an industrial organic waste product and the establishment of relationships between its composition, interfacial behavior, and flotation activity. The practical significance of the research lies in the creation of a more accessible, resource-efficient, and effective flotation collector for processing finely disseminated sulfide ores. Unlike traditional xanthates synthesized from standard alcohols, this study proposes a method for producing a multicomponent xanthate collector from distillery waste, thereby providing a wider range of hydrophobic properties and enhanced adsorption onto mineral surfaces.

## 2. Materials and Methods

### 2.1. Materials

Fusel oil obtained as a by-product of ethanol production at the Talgar Distillery (Talgar, Kazakhstan) was used as the starting material. The qualitative and quantitative composition of the fusel oil was determined by gas chromatography-mass spectrometry (GC-MS) using an Agilent 7890A instrument equipped with a 5975C mass-selective detector (Agilent Technologies, Santa Clara, CA, USA). The reagents used were potassium hydroxide (KOH, analytical grade; Sigma-Aldrich, St. Louis, MO, USA) and carbon disulfide (CS_2_, analytical grade; Sigma-Aldrich, St. Louis, MO, USA). All reagents were used without further purification.

Monomineralic samples of chalcopyrite (CuFeS_2_), galena (PbS), sphalerite (ZnS), and pyrite (FeS_2_) were used as the objects of study. The phase composition of the initial mineral samples was investigated using X-ray diffraction (XRD) analysis. Measurements were performed on a D8 Advance X-ray diffractometer (ruker AXS GmbH, Karlsruhe, Germany) using Cu Kα radiation.

### 2.2. Preparation of a Multicomponent Xanthate Collector

The multicomponent collector was synthesized by reacting the mixture of alcohols found in fusel oil with potassium hydroxide and carbon disulfide. The reaction was carried out at a temperature between −5 and 0 °C under constant stirring. The molar ratio of the components was an alcohol mixture of CS_2_: KOH = 1: 0.6: 0.6. The process duration was 2 h.

Given the multicomponent nature of the alcohol mixture, the synthesis of the xanthate collector can be represented by generalized reaction schemes (1) and (2), illustrating the simultaneous interaction of various alcohols with potassium hydroxide and carbon disulfide to form the corresponding alkyl xanthates.

(1) −OH+KOH→R−OK+HOH

(2) $$R-\mathrm{OK}+CS_{2}\to R-\mathrm{OC}\left(S\right)\mathrm{SK}$$

### 2.3. Calculation of the Hydrophilic-Lipophilic Balance

Calculation and evaluation of the hydrophilic–lipophilic balance (HLB) of the synthesized xanthates. Griffin’s formula (3) was used to determine the *HLB*:

(3) $$\mathrm{HLB}=20x\frac{M_{\mathrm{hydrophilic}}}{M_{\mathrm{molecule}}}$$

where

*M_hydrophili_*_c_ is the molecular mass of the hydrophilic part of the molecule,

*M_molecule_* is the total molecular mass of the entire molecule.

For a multicomponent system, the calculated HLB value was determined taking into account the mass fraction of each xanthate component present (4):

(4) $${\mathrm{HLB}}_{\mathrm{MX}}=\sum_{i=1}^{n}w_{i}{\mathrm{HLB}}_{i}$$

where *w_i_* is the mass fraction of the i-th component, and *HLB_i_* is its calculated *HLB_MX_* value.

### 2.4. Flotation Tests

Flotation experiments were carried out using the FML-0.1 laboratory flotation machine (JSC Research and Engineering Corporation “Mekhanobr-Tekhnika”, St. Petersburg, Russia), which ensures stable pulp agitation and uniform distribution of air bubbles, thereby creating favorable hydrodynamic conditions for effective interaction between mineral particles and air bubbles during the flotation process. The pulp pH was maintained at 9 throughout flotation. The tests were carried out under two conditions: a baseline condition using potassium amyl xanthate (PAX) and a condition using a synthesized multicomponent xanthate collector at concentrations of 2.5, 5.0, and 7.5 mg/L. Reagent X-133 was used as the frother at a dosage of 15 mg/L. The flotation duration was 30, 60, 120, 150, and 180 s. The air flow rate was maintained at 10 L/min, and the impeller rotation speed was 30 Hz. For each experimental condition, three independent flotation tests were conducted *(n = 3*). Shows the arithmetic mean recovery values, with error bars representing ± one sample standard deviation (SD), calculated based on the results of the three independent tests. In the flotation experiment, a 2 g sample of the mineral was used with 100 mL of water, corresponding to a solids mass fraction in the pulp of approximately 1.96%. The purity of the minerals used exceeded 95%. First, the collector was added to the pulp, followed by a 2 min conditioning period. Then, the frother X-133 was added, and conditioning continued for another 1 min, after which the flotation process was carried out.

The recovery of the froth product was calculated using the following formula (5):

Recovery = (mₖ/m_0_) × 100% (5)

where mₖ is the mass of the dry froth product (in grams) and m_0_ is the initial mass of the mineral sample (2 g).

### 2.5. Determination of the Zeta Potential

To assess the influence of flotation collectors on the electrokinetic properties of the surface of sulfide minerals, the zeta potential of chalcopyrite, galena, sphalerite, and pyrite was determined. Measurements were carried out using a Photocor Compact instrument by the electrophoresis method within a pH range of 2 to 10. The pH of the suspensions was adjusted by adding 0.1 M solutions of HCl and NaOH.

Suspensions of the original single-mineral samples in deionized water were investigated, as well as samples following interaction with potassium amylxanthate (PAX) and a multicomponent xanthate collector (MX). The concentrations of PAX and MX were 5 mg/L. Measurements were carried out at pH 2, 4, 6, 8, and 10. Each zeta potential value was calculated as the average of three parallel measurements (n = 3).

### 2.6. FTIR Spectroscopy of the Chalcopyrite Surface

Fourier transform infrared (FTIR) spectroscopy was used to investigate changes in the functional groups on the surface of chalcopyrite following interaction with a multicomponent xanthate collector (MX). Spectra were recorded on an FT/IR-6X spectrometer (JASCO, Japan) in the wavenumber range 4000–350 cm^−1^. The chalcopyrite was treated with a multicomponent xanthate at a concentration of 5 mg/L and a pH of 9, after which the solid phase was separated by filtration, dried at room temperature, and stored in a desiccator until analysis.

Samples for FTIR analysis were prepared by KBr pressing. Approximately 50 mg of the powdered sample was thoroughly mixed with spectroscopically pure KBr in a mass ratio of 1:100, after which the resulting mixture was pressed to form transparent pellets. The spectrum of pure KBr was used as the background spectrum.

The spectra obtained were processed and interpreted using Spectra Manager software, version 2.5, and the KnowItAll spectral database, with reference to relevant literature data.

### 2.7. X-Ray Photoelectron Spectroscopy of the Chalcopyrite Surface

X-ray photoelectron spectroscopy (XPS) was used to investigate the elemental composition and chemical state of the chalcopyrite surface before and after interaction with a multi-component xanthate collector (MX). Measurements were carried out on a NEXSA spectrometer (Thermo Fisher Scientific, Waltham, MA, USA), equipped with a monochromatic Al Kα source with a radiation energy of 1486.6 eV. The source operating parameters were 25 kV and 10 mA. Spectra were recorded under high-vacuum conditions.

To prepare the initial sample, 5.0 g of chalcopyrite was dispersed in 100 mL of deionized water. The pH of the suspension was adjusted to 9 using a NaOH solution. To investigate the effect of the collector, the chalcopyrite was treated with MX at a concentration of 5 mg/L. After 15 min of conditioning, the suspension was filtered. The resulting filter cake was not washed with deionized water and was dried directly at room temperature, then stored in a desiccator until XPS analysis.

Survey spectra were recorded for both the initial and MX-treated chalcopyrite, as well as high-resolution spectra in the Cu 2p, S 2p, Fe 2p, O 1s, and C 1s binding energy regions. The binding energy scale was calibrated relative to the C 1s peak at 284.80 eV. The spectra obtained were processed using OriginPro 2026 (version 10.35, OriginLab).

## 3. Results and Discussion

### 3.1. Characterization of the Raw Material and Synthesis Products

The chromatographic analysis results showed that the fusel oil is a multicomponent mixture of alcohols with varying molecular structures. The main components are isoamyl alcohol (55.9%) and ethanol (29.7%); 1-pentanol (7.8%), isobutanol (4.6%), propanol (1.0%), phenylethyl alcohol (1.0%), and other components are present in smaller amounts.

**Figure 1:**
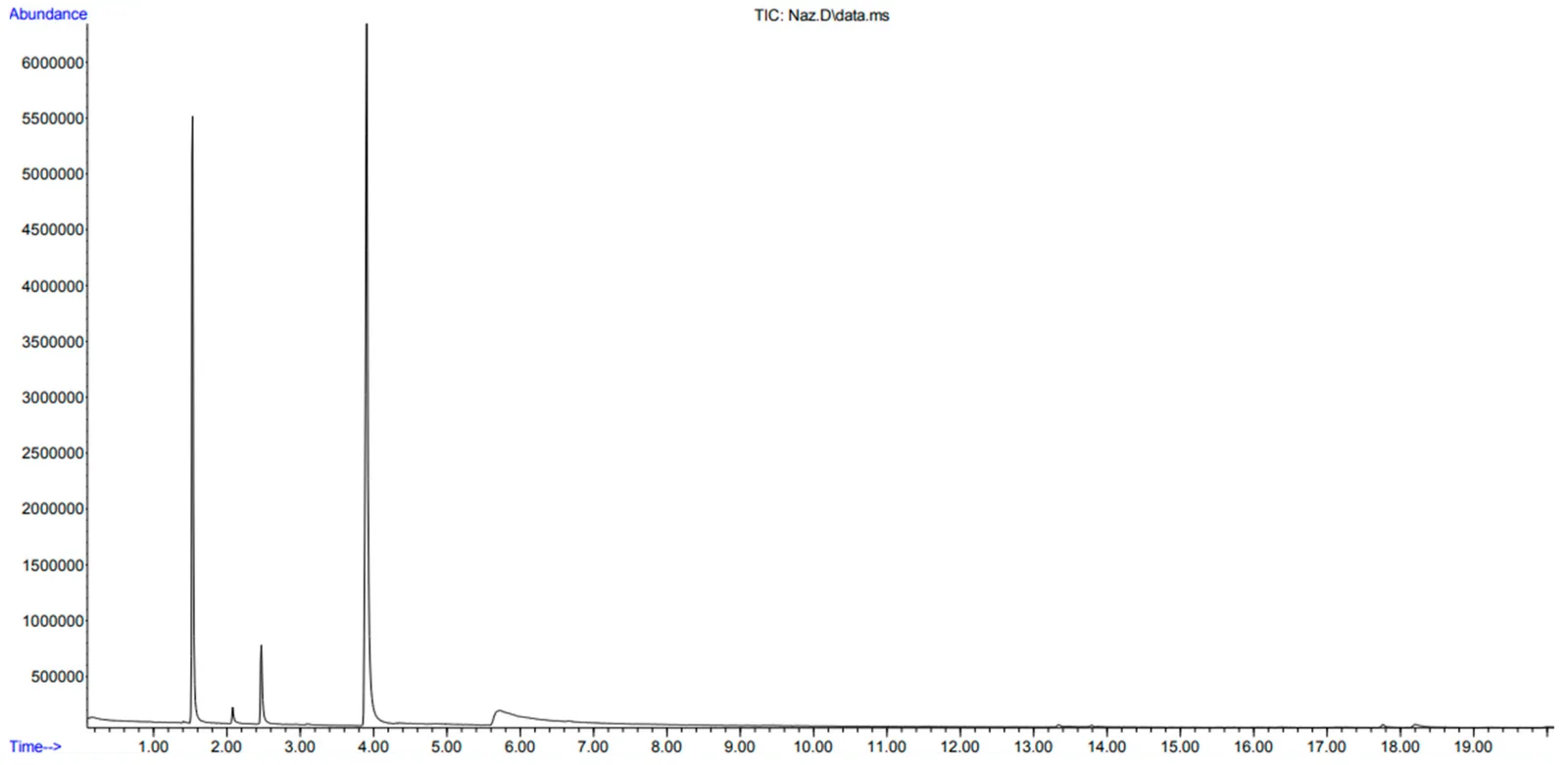
Chromatogram of fusel oil.

The analysis results are presented in Table 1 and Figure 1.

**Figure A1:**
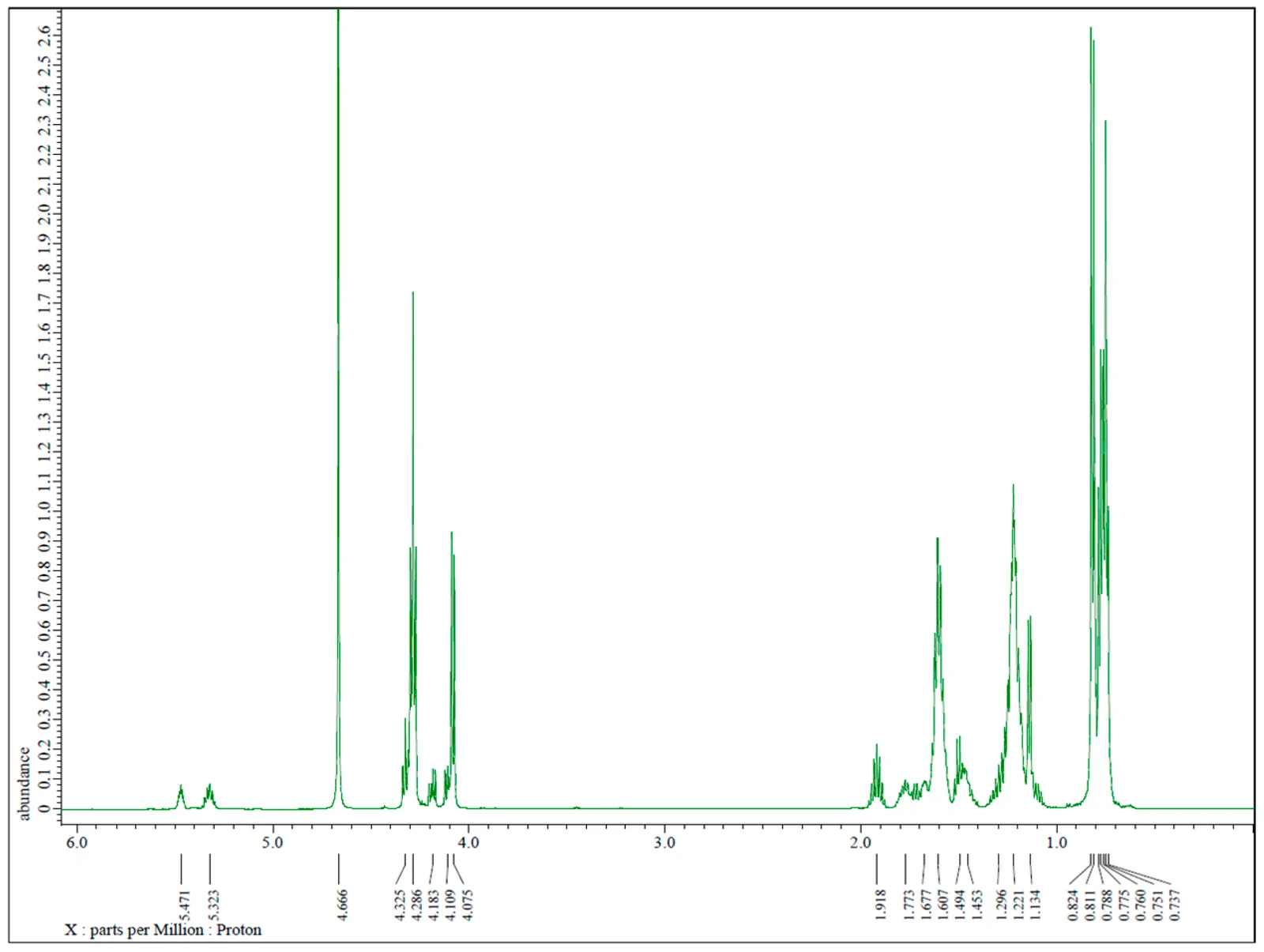
^1^H NMR spectrum of the multicomponent xanthate (MX) synthesized from fusel oil (solvent: D_2_O).

**Figure A2:**
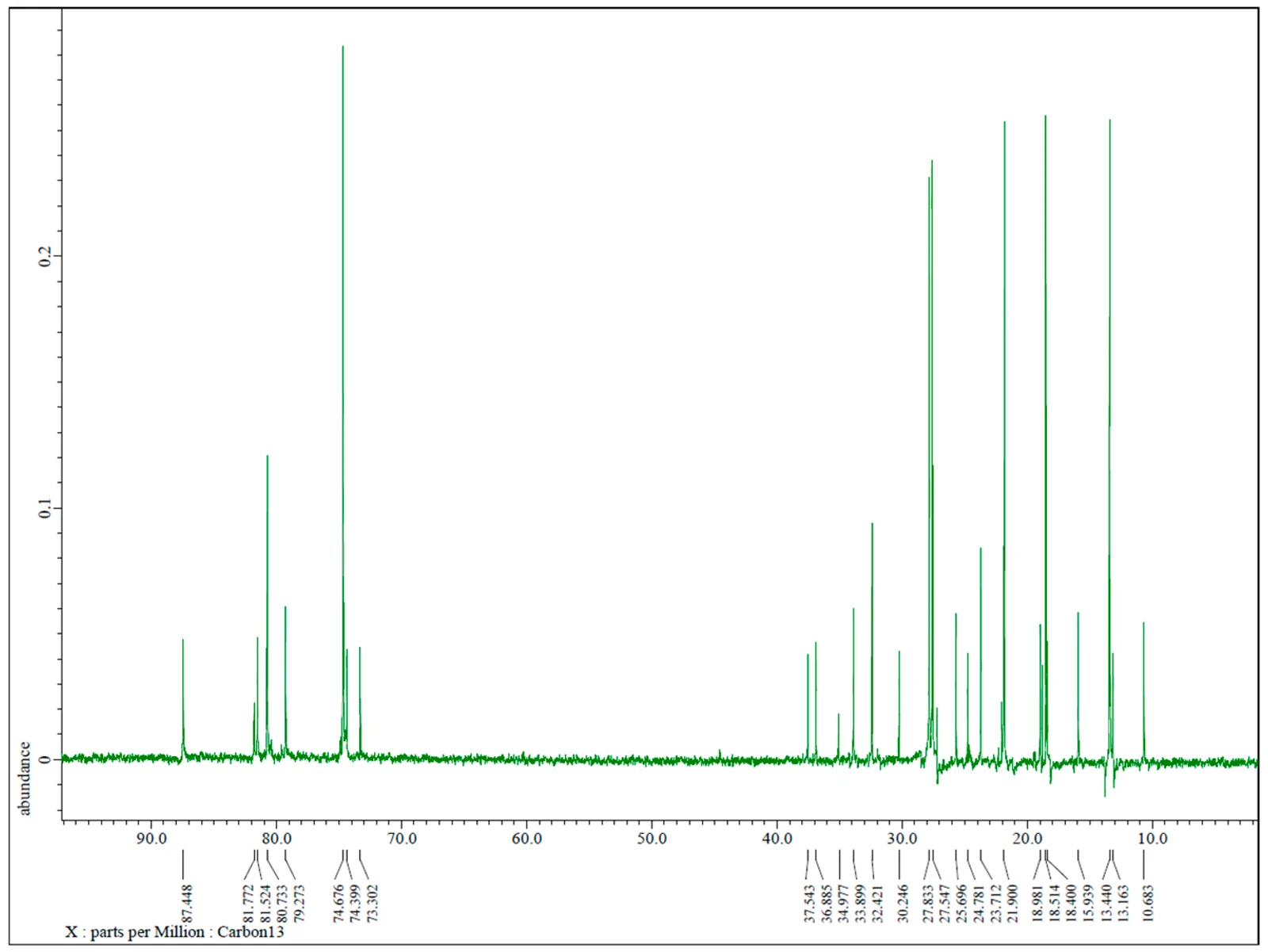
NMR spectrum ^13^C of the multicomponent xanthate (MX) synthesized from fusel oil (solvent D_2_O).

The synthesized multicomponent xanthate (MX) was characterized using ^1^H and ^13^C nuclear magnetic resonance (NMR) spectroscopy. Spectra were recorded on a JNM-ECA 500 (JEOL) NMR spectrometer using D_2_O as the deuterated solvent. In the ^1^H NMR spectrum, major signals were observed in the ranges of δ 4.666–4.075 ppm (OCH_2_), 1.918–1.607 ppm (β-/γ-CH_2_), 1.494–1.134 ppm (aliphatic CH_2_), and 0.824–0.737 ppm (terminal CH_3_). In the ^13^C NMR spectrum, signals were observed in the ranges δ 87.448–73.302, 37.843–21.900, and 18.981–10.683 ppm, corresponding to oxygen-bearing carbon centers and aliphatic CH_2_ and CH_3_ groups, respectively. The full ^1^H and ^13^C NMR spectra of the multicomponent xanthate are presented in Appendix A (Figure A1 and Figure A2).

Based on the composition of the alcohol mixture, the theoretical product yield was calculated using the proposed mechanism for the reaction of the alcohols with potassium hydroxide and carbon disulfide, which leads to the formation of xanthate compounds (1) and (2). The reaction scheme involves the neutralization of the alcohols to form alkoxides, followed by their reaction with CS_2_ to yield the corresponding potassium xanthate salts (6) and (7):

(6) $$R-\mathrm{OH}+CS_{2}+\mathrm{KOH}\to \;R-\mathrm{OC}\left(S\right)\mathrm{SK}+\mathrm{HOH}$$

(7) $$\left\{\begin{array}{c} C_{2}H_{5}-\mathrm{OH}\\ {(\mathrm{CH}}_{3}{)}_{2}\mathrm{CH}{\mathrm{CH}}_{2}-\mathrm{OH}\\ {(\mathrm{CH}}_{3}{)}_{2}\mathrm{CH}{(\mathrm{CH}}_{2}{)}_{2}-\mathrm{OH}\\ {\mathrm{CH}}_{3}{(\mathrm{CH}}_{2}{)}_{4}-\mathrm{OH} \end{array}\right.+4CS_{2}+4\mathrm{KOH}\to \left\{\begin{array}{c} C_{2}H_{5}-OC\left(S\right)\mathrm{SK}\\ {(\mathrm{CH}}_{3}{)}_{2}\mathrm{CH}{\mathrm{CH}}_{2}-OC\left(S\right)\mathrm{SK}\\ {(\mathrm{CH}}_{3}{)}_{2}\mathrm{CH}{(\mathrm{CH}}_{2}{)}_{2}-OC\left(S\right)\mathrm{SK}\\ {\mathrm{CH}}_{3}{(\mathrm{CH}}_{2}{)}_{4}-OC\left(S\right)\mathrm{SK} \end{array}\right.+4\mathrm{HOH}$$

To calculate the theoretical yield of xanthates, a scheme based on the alcohols identified via chromatographic analysis of fusel oil was used. The mixture consisted of ethanol (29.7%), isobutyl alcohol (4.6%), isoamyl alcohol (55.9%), and amyl alcohol (7.8%).

We calculated the theoretical mass of the product, given that each alcohol yields 1 mole of the corresponding xanthate (Table 2).

Assuming complete conversion of the alcohols and disregarding losses, the theoretical yield of the potassium xanthate mixture is 109.67 g.

Calculated HLB values for individual xanthates and their mixtures are presented in Table 3.

Griffin’s equation was originally developed for non-ionic surfactants, and its application to ionic xanthates requires caution. In this study, this approach was used to obtain an approximate descriptor based on the composition of the substance, which reflects the relative contribution of the hydrophilic and lipophilic parts of the collector. An example of the application of this approach to xanthogenates, including potassium amylxanthogenate, is provided in [31]. However, this calculation does not explicitly take into account factors such as ionic dissociation, hydration, the influence of counterions, or solution conditions. Consequently, the calculated HLB (hydrophilic–lipophilic balance) value should be regarded as a nominal structural estimate, rather than as an experimentally determined measure of water solubility, adsorption capacity, or flotation selectivity.

### 3.2. Mineral Characterization

**Figure 2:**
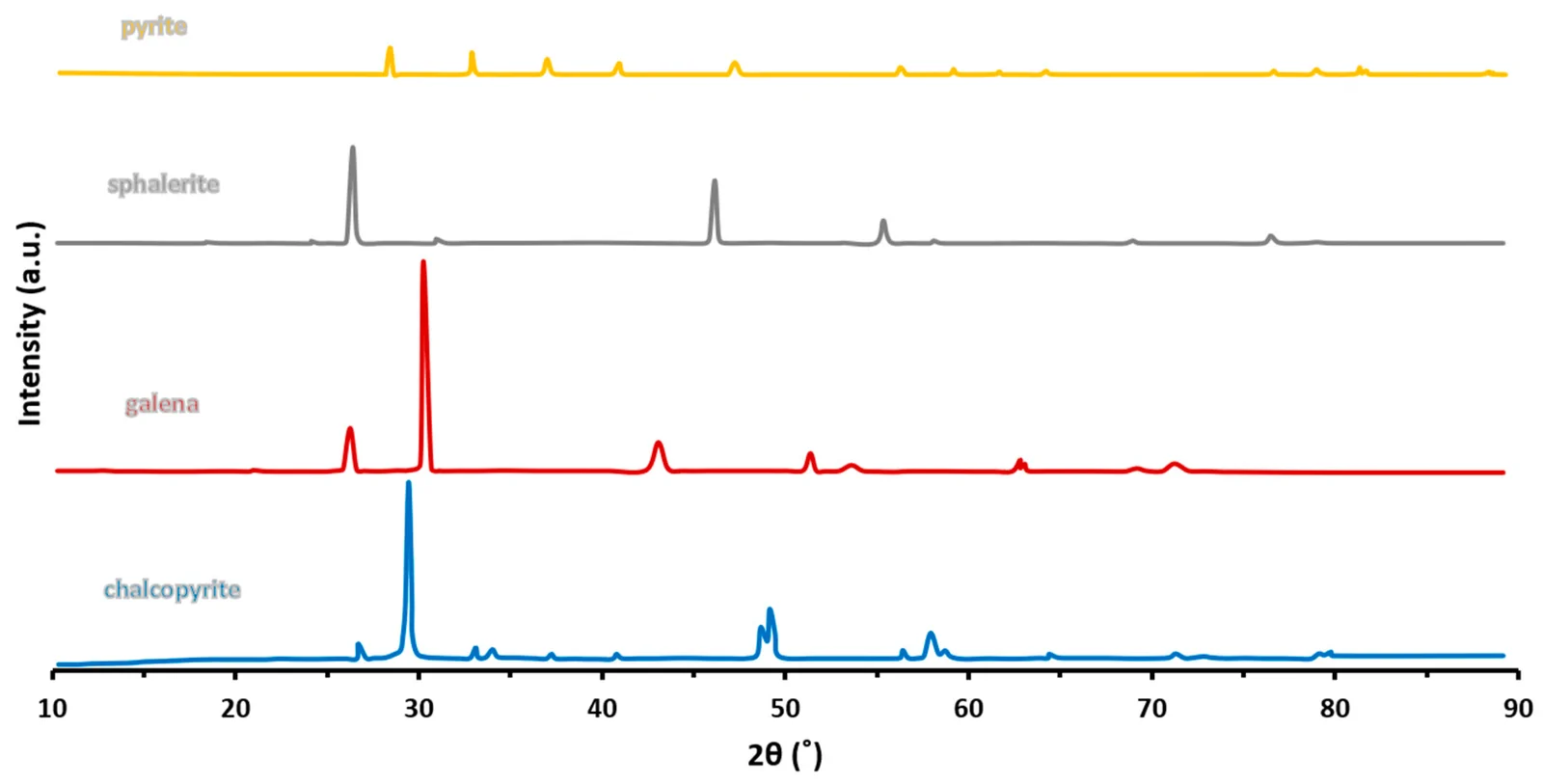
X-ray diffraction patterns of the initial samples of chalcopyrite, galena, sphalerite, and pyrite. Reference patterns: PDF 01-083-0983, PDF 00-005-0592, PDF 00-005-0566, and PDF 00-042-1340, respectively.

The obtained diffractograms were used to identify the major crystalline phases of the sulfide minerals under study (Figure 2). The characteristic diffraction peaks were assigned to chalcopyrite (CuFeS_2_), galena (PbS), sphalerite (ZnS), and pyrite (FeS_2_) by comparison with the reference patterns PDF 01-083-0983, PDF 00-005-0592, PDF 00-005-0566, and PDF 00-042-1340, respectively. For chalcopyrite, the most intense peak was observed at 2θ ≈ 29.45°, with additional reflections at approximately 33.96°, 48.71°, 49.12°, 57.93°, 58.63°, 71.28°, and 78.95°. These results support the identification of the principal crystalline phases in the mineral samples used for the flotation experiments.

### 3.3. Effect of MX Concentration and Particle Size on Flotation Kinetics

**Figure 3:**
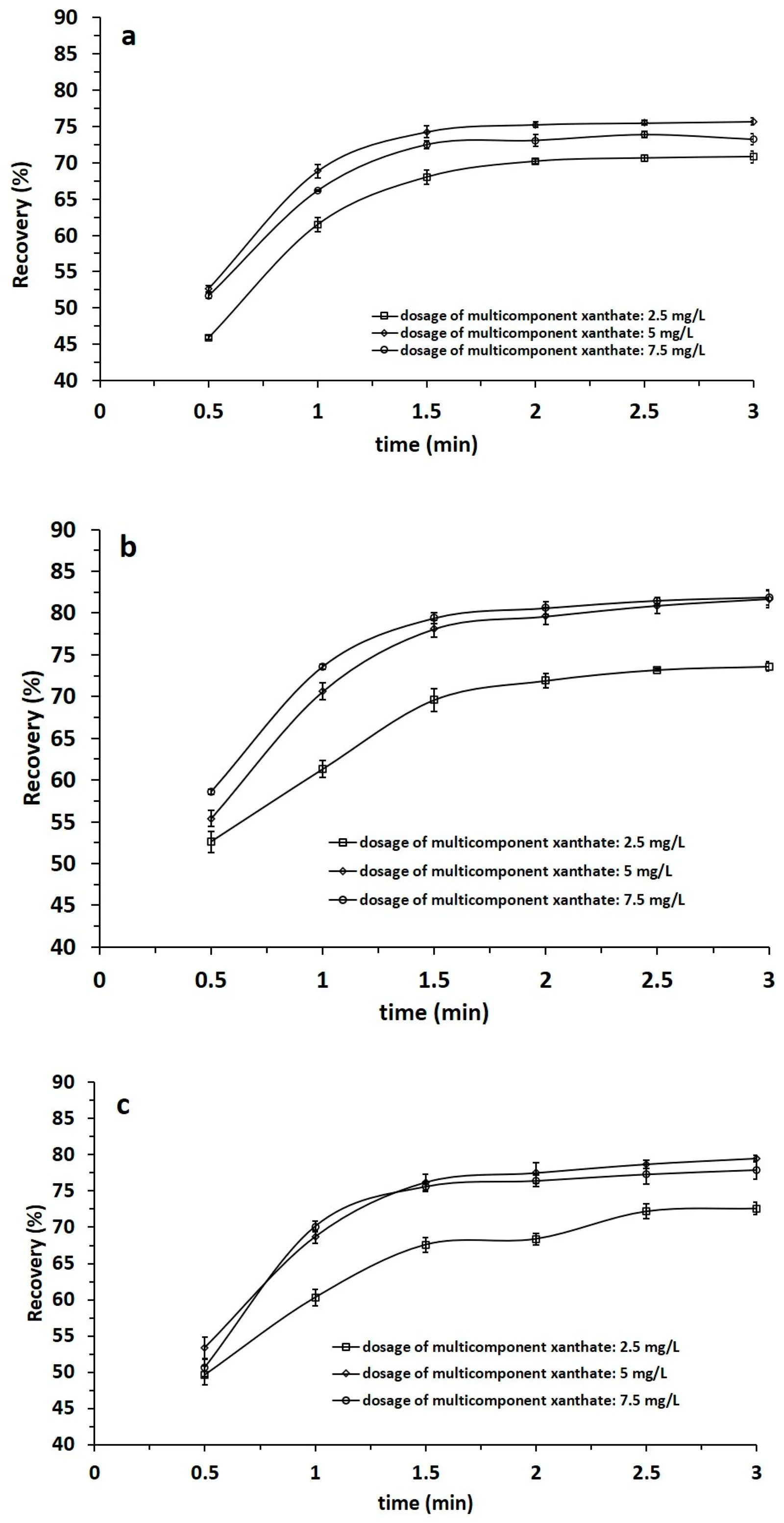

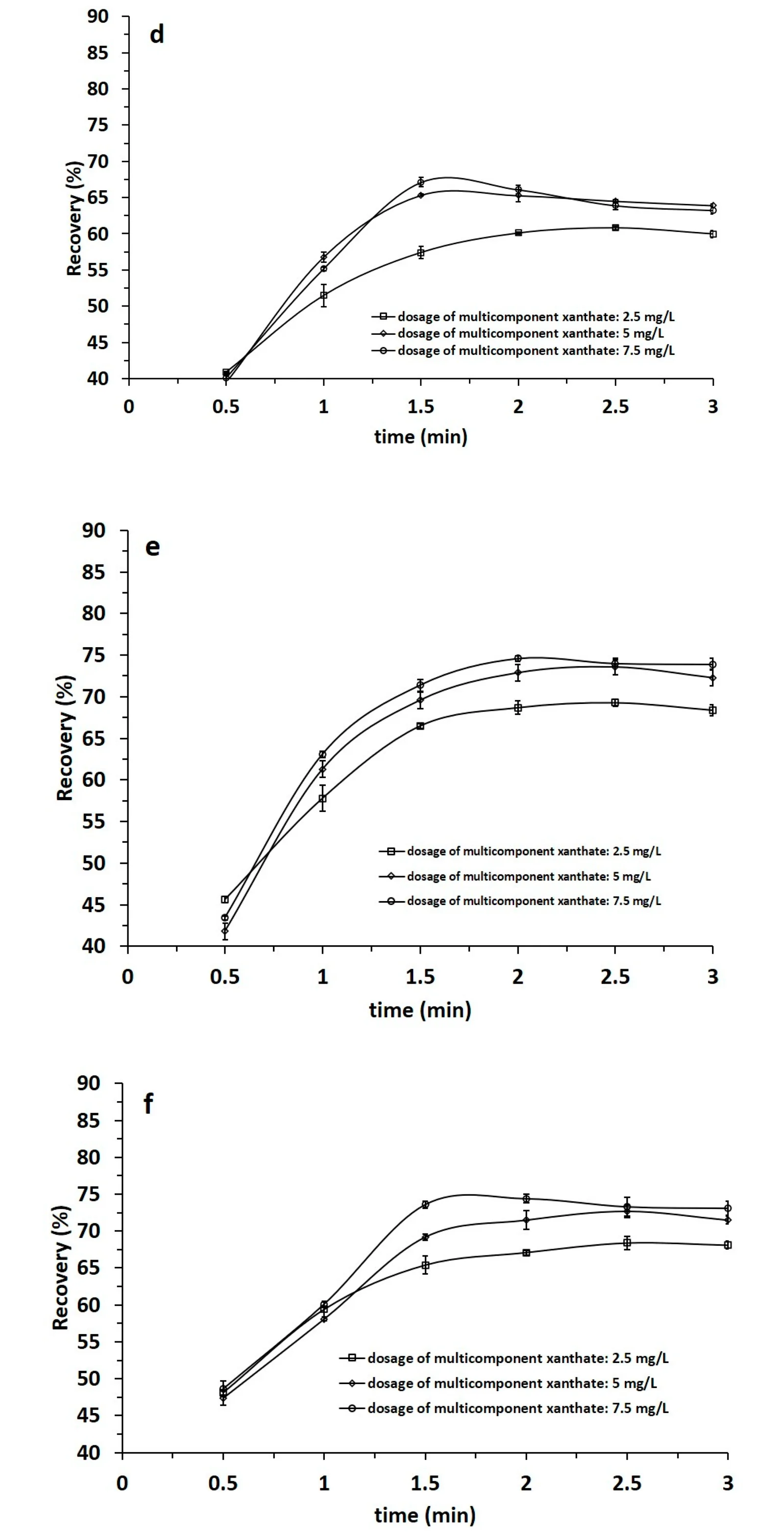

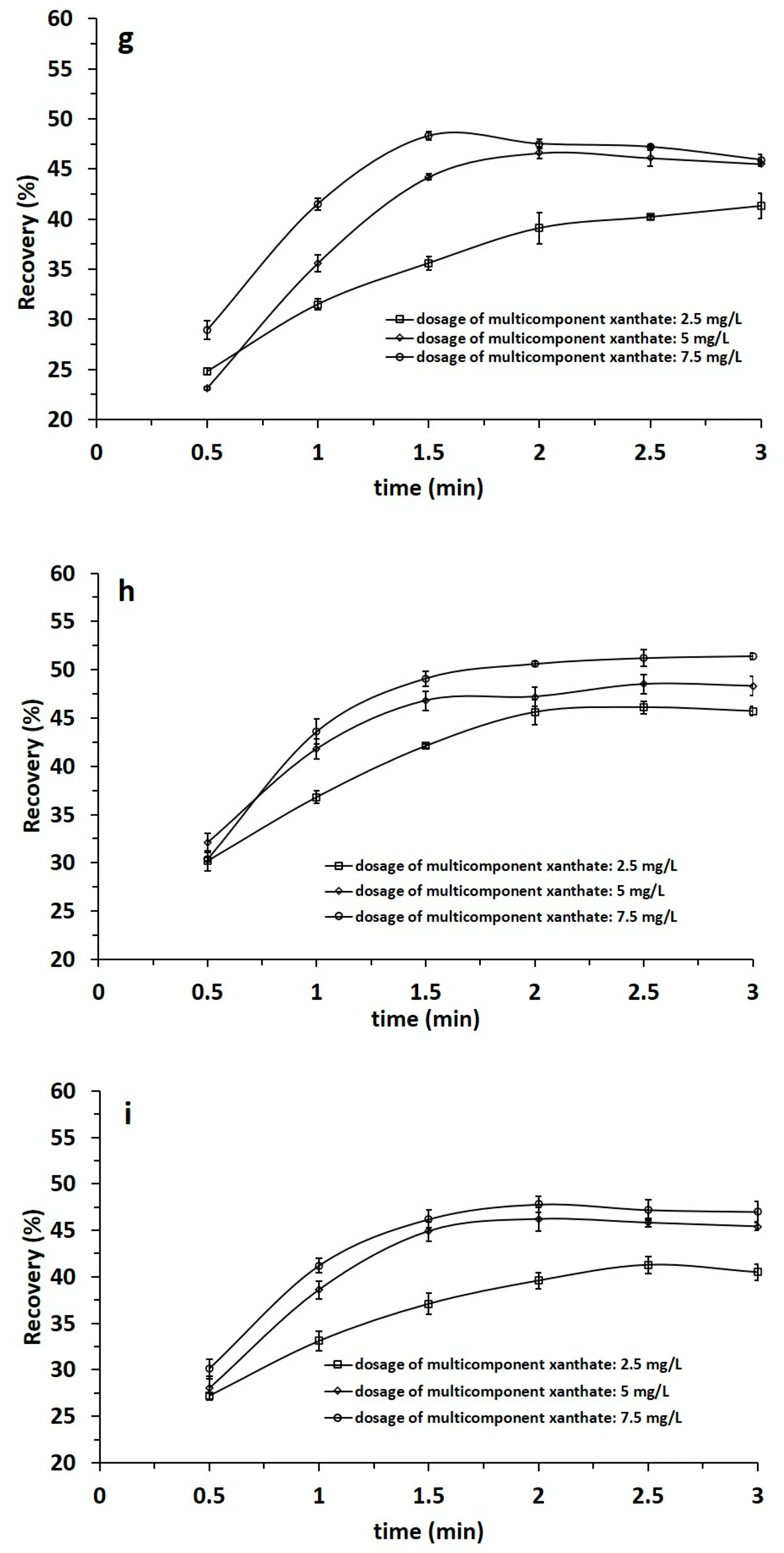

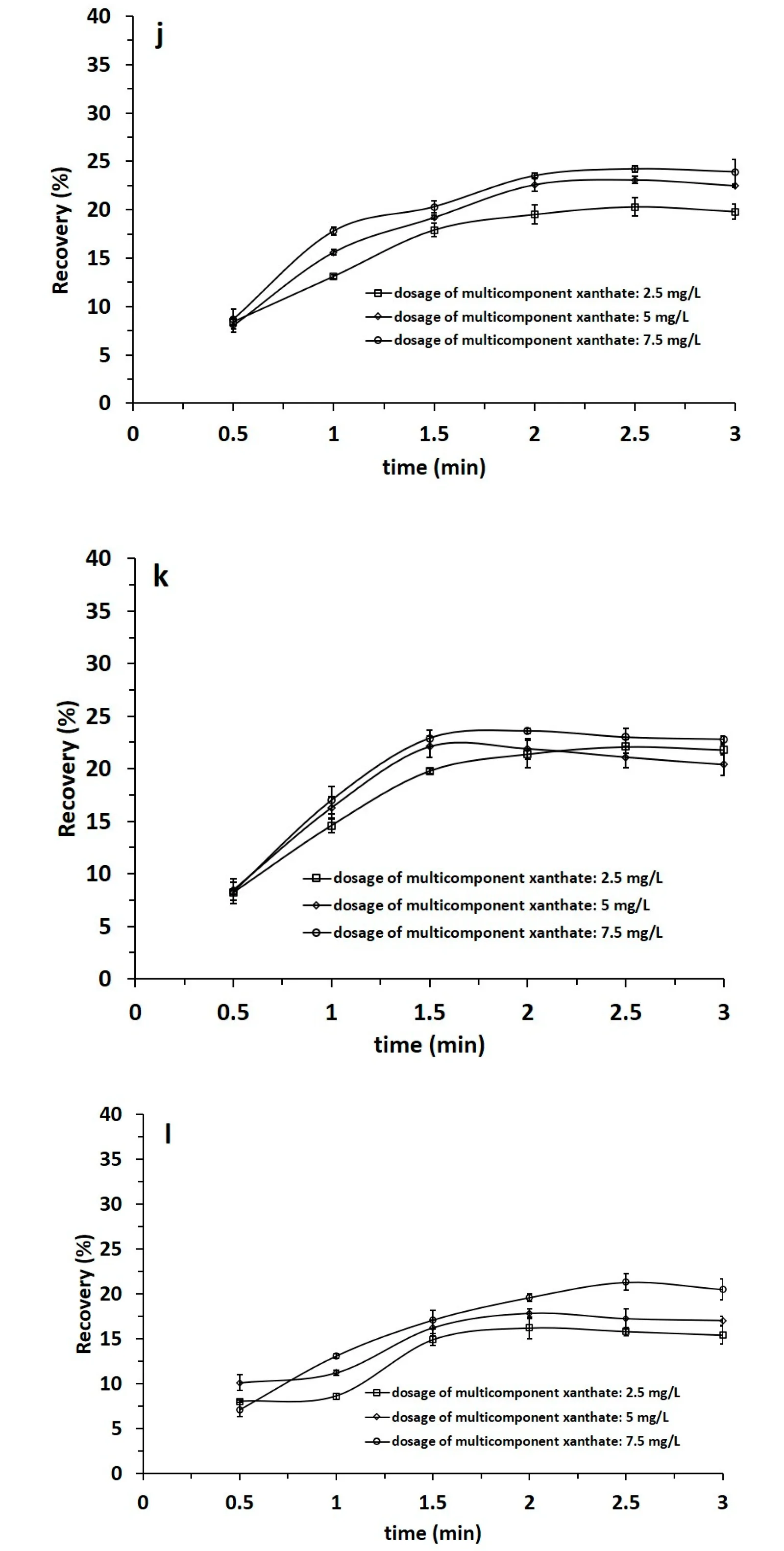
Kinetic curves for the separation of chalcopyrite (**a**–**c**), galena (**d**–**f**), sphalerite (**g**–**i**) and pyrite (**j**–**l**) at various concentrations of the multicomponent xanthate collector (MX) 2.5; 5.0 and 7.5 mg/L: (**a**,**d**,**g**,**j**)—−0.100 + 0.074 mm fraction; (**b**,**e**,**h**,**k**)—−0.074 + 0.040 mm; (**c**,**f**,**i**,**l**)—−0.040 + 0 mm.

The kinetic curves for the recovery of chalcopyrite, galena, sphalerite, and pyrite at MX concentrations of 2.5, 5.0, and 7.5 mg/L are shown in Figure 3. The results obtained show that the flotation behavior of the multicomponent collector is determined by the nature of the sulfide mineral, as well as by particle size and reagent concentration. For all the minerals studied, the most pronounced increase in recovery was observed predominantly during the first 1.5–2 min of flotation, after which the curves approached a plateau. The highest recovery values were characteristic of chalcopyrite and galena, whilst sphalerite and, in particular, pyrite exhibited significantly lower flotability.

For chalcopyrite, a marked effect of MX concentration was observed across all particle size classes studied. For the coarse fraction (−0.100 + 0.074 mm), recovery after 3 min of flotation was 70.83%, 75.7%, and 73.23% at MX concentrations of 2.5; 5.0 and 7.5 mg/L, respectively (Figure 3a). Thus, increasing the collector concentration from 2.5 to 5.0 mg/L led to a 4.87% increase in recovery, whilst a further increase in concentration to 7.5 mg/L did not yield any additional benefit. A similar trend was observed for the −0.040 + 0 mm fraction, where the final recovery was 72.6%, 79.5%, and 77.9%, respectively (Figure 3c). The maximum chalcopyrite recovery of 81.5% was achieved for the −0.074 + 0.040 mm fraction at an MX concentration of 7.5 mg/L after just 2.5 min of flotation; after 3 min, it stood at 81.9% (Figure 3b). Overall, the results indicate high flotation activity of MX towards chalcopyrite, with the optimum concentration depending on particle size.

For galena, an increase in the MX concentration also contributed to accelerated flotation and an increase in final recovery; however, the effect was most pronounced for the intermediate and fine fractions. For the −0.100 + 0.074 mm fraction, values of 59.973%, 63.9%, and 63.23% were obtained after 3 min at 2.5; 5.0 and 7.5 mg/L, respectively (Figure 3d). For the −0.074 + 0.040 mm fraction, the final recovery increased from 68.4% at 2.5 mg/L to 72.3% at 5.0 mg/L and 73.9% at 7.5 mg/L (Figure 3e). The highest value for galena was obtained for the −0.040 + 0 mm fraction: at 7.5 mg/L, recovery reached 74.4% after 2 min, after which it remained virtually unchanged at 73.1% after 3 min (Figure 3f). The fact that the curves level off after approximately 2 min indicates that the majority of the floatable galena is recovered at an early stage of the process.

For sphalerite, the recovery values were significantly lower than for chalcopyrite and galena. For the coarse fraction (−0.100 + 0.074 mm), recovery after 3 min was 41.3; 45.5 and 45.9% at MX concentrations of 2.5, 5.0, and 7.5 mg/L, respectively (Figure 3g). For the intermediate fraction, increasing the MX concentration from 2.5 to 7.5 mg/L raised the final separation rate from 45.7 to 51.4% (Figure 3h). For the fine fraction, the corresponding values were 40.5; 45.4 and 47.0% (Figure 3i). Consequently, an increase in the MX concentration has a positive effect on the flotation of sphalerite; however, the absolute recovery values remain significantly lower than those for chalcopyrite and galena.

Pyrite exhibited the lowest flotability in the presence of MX. For the coarse fraction (−0.100 + 0.074 mm), after 3 min the recovery was 19.8; 22.5 and 23.9% at 2.5; 5.0 and 7.5 mg/L, respectively (Figure 3j). For the −0.074 + 0.040 mm fraction, the values ranged from 21.8 to 22.8% (Figure 3k), whilst for the finest fraction, −0.040 + 0 mm, they ranged from 15.4 to 20.5% (Figure 3l). The maximum pyrite recovery did not exceed approximately 24%, despite an increase in the collector concentration.

### 3.4. Surface Properties

**Figure 4:**
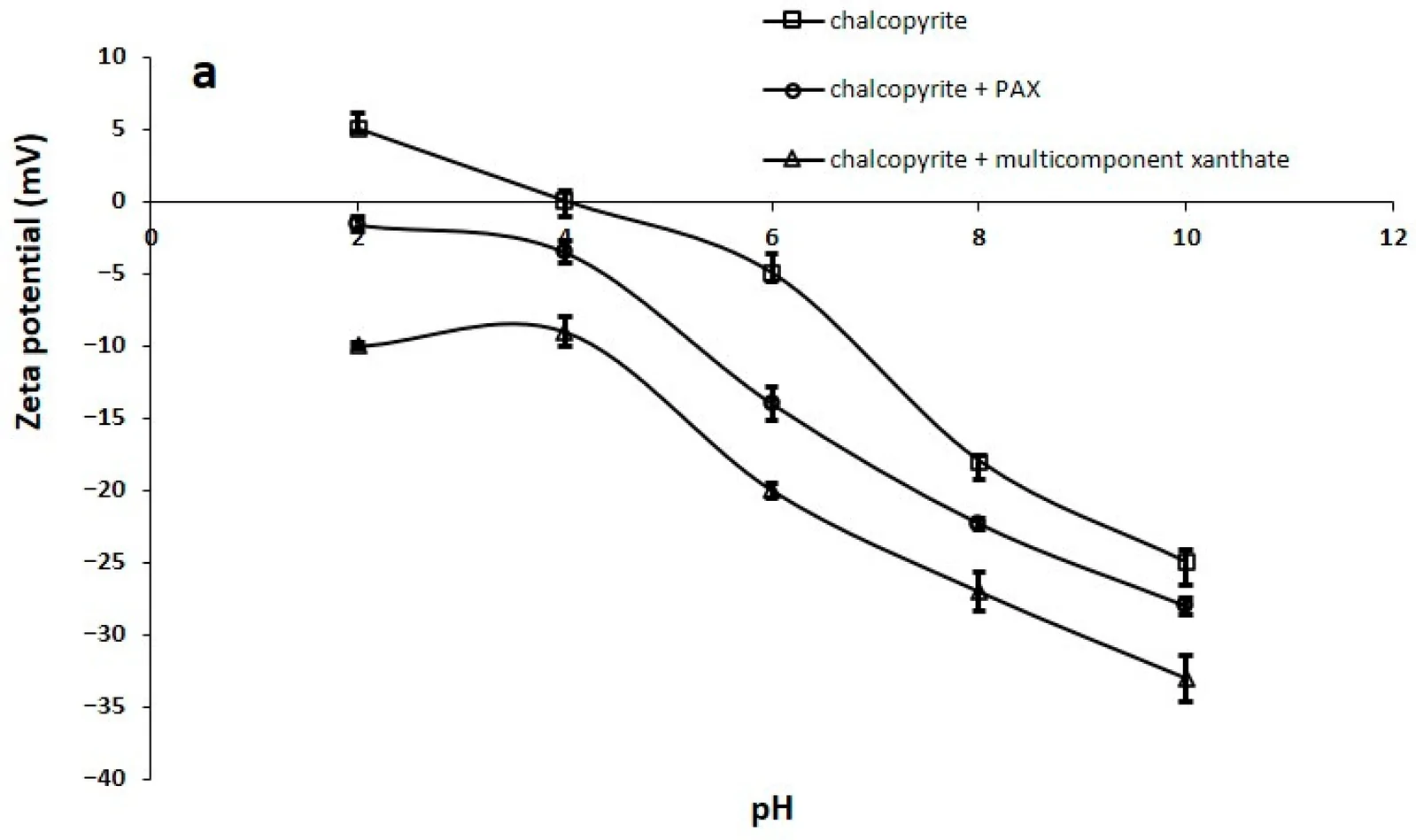

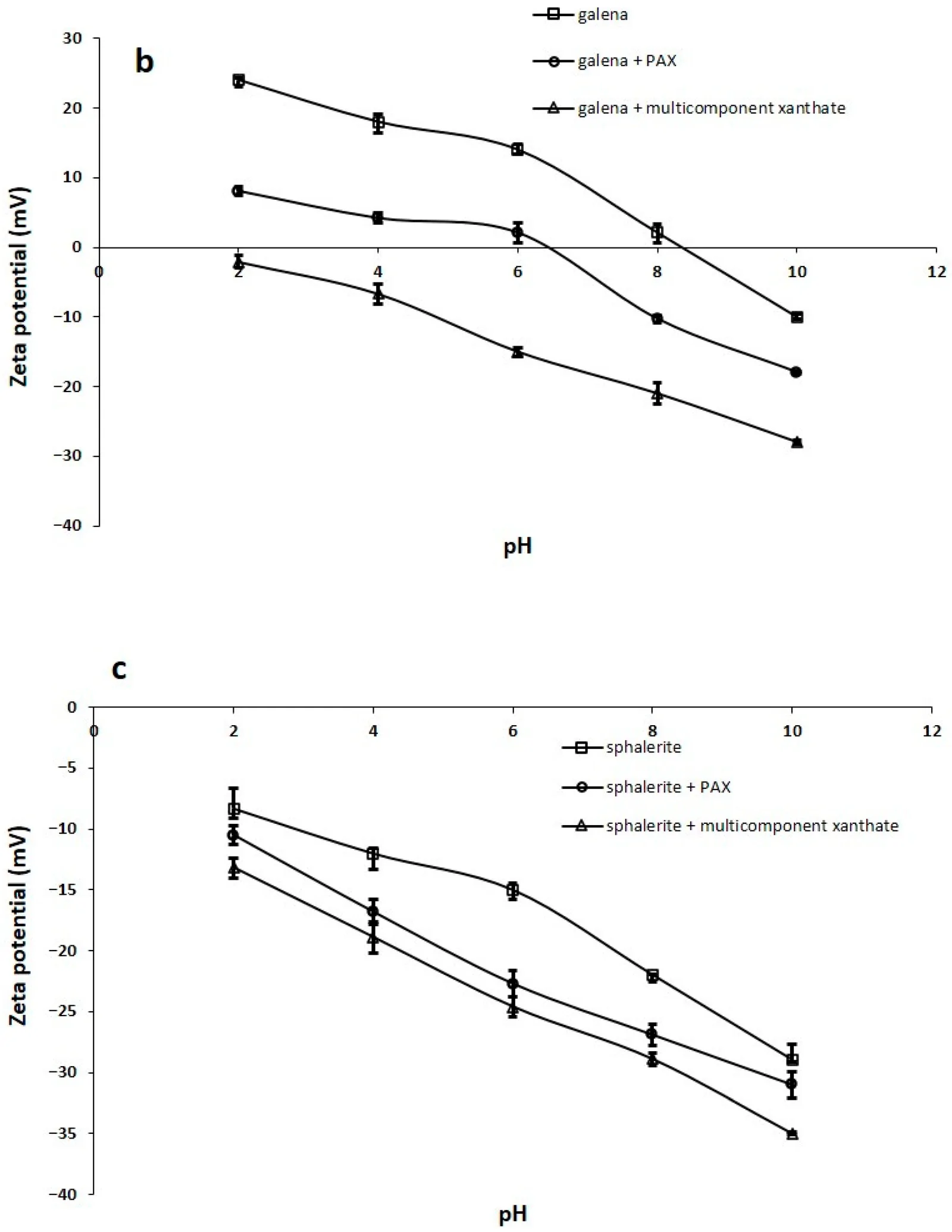

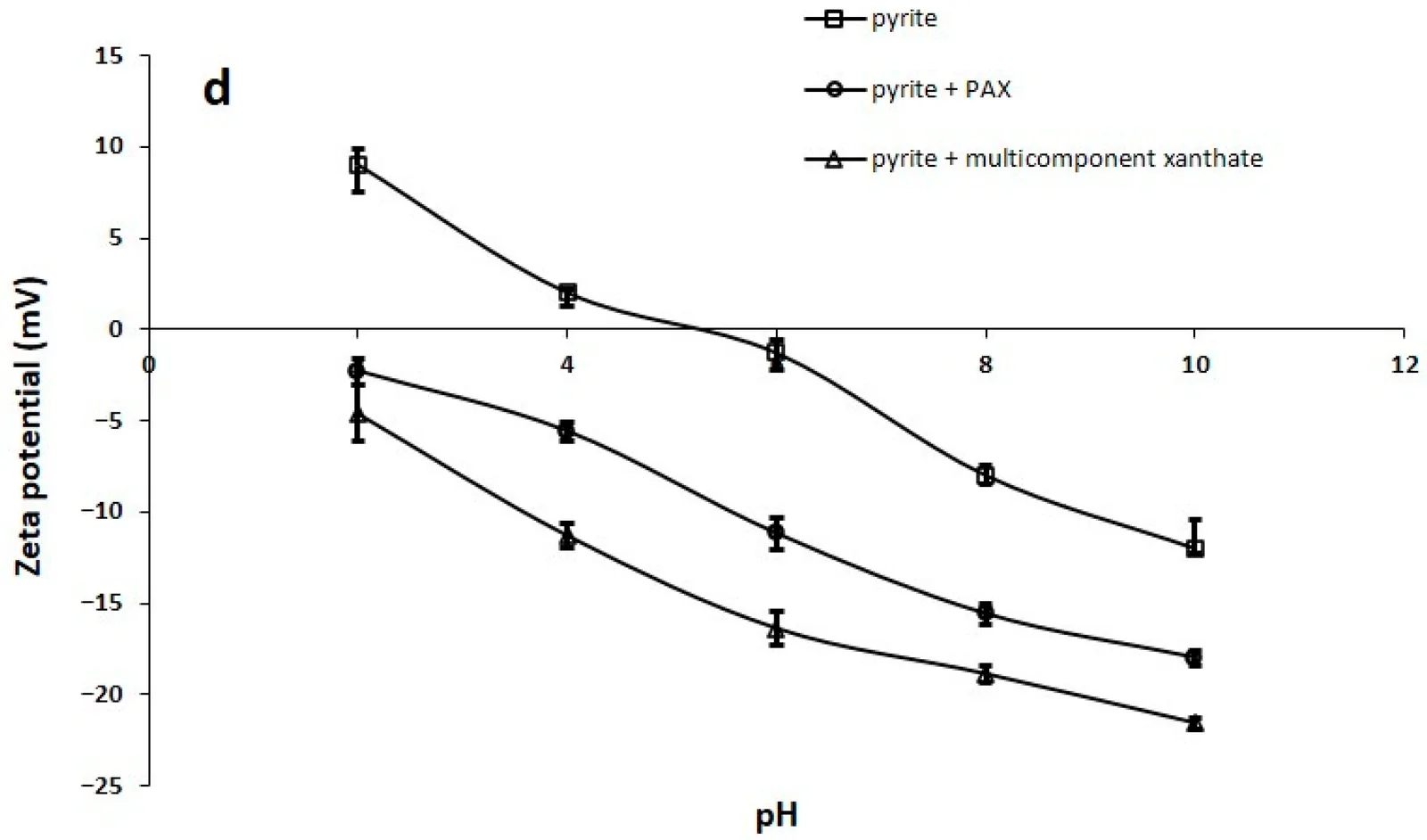
Effect of pH on the zeta potential of (**a**) chalcopyrite; (**b**) galena; (**c**) sphalerite; (**d**) pyrite in the absence of a collector and following treatment with PAX and a multicomponent xanthate.

The effect of pH on the zeta potential of chalcopyrite is shown in Figure 4a. As the pH increases, the zeta potential of the original chalcopyrite shifts from +5.0 mV at pH 2 to −25.0 mV at pH 10. Treatment with PAX results in a further shift in the potential into the negative region, whilst the multicomponent xanthate has the most pronounced effect: the values range from −10.0 mV at pH 2 to −33.0 mV at pH 10. At pH 6, the zeta potential is approximately −5, −14, and −20 mV for the initial chalcopyrite, chalcopyrite with PAX, and the multicomponent xanthate, respectively. The more pronounced negative shift in the presence of multicomponent xanthate indicates a significant change in the interfacial electrical properties of the surface and is consistent with the adsorption of xanthate components onto the active sites of chalcopyrite. A similar change in surface charge upon interaction with xanthate collectors has been reported previously [1,3,8,11,12,13,14]. The results obtained indicate a more pronounced interaction between the developed multicomponent collector and the chalcopyrite surface compared with PAX.

Changes in the ζ-potential of sulfide minerals as a function of pH allow the influence of collectors on the state of the mineral–solution interface to be assessed. As can be seen from Figure 4, the treatment of chalcopyrite, galena, sphalerite, and pyrite with both potassium amylxanthate (PAX) and a multicomponent xanthate collector (MX) leads to a shift in the ζ-potential into the negative region relative to the untreated minerals. Furthermore, across the entire pH range studied, the effect of MX is more pronounced than that of PAX, indicating a more significant modification of the surface’s electrical properties in the presence of the multicomponent collector. As the pH increases, the zeta potential of the initial chalcopyrite shifts from +5.0 mV at pH 2 to −25.0 mV at pH 10. Treatment with PAX leads to a further shift in the potential into the negative region, whilst the multicomponent xanthate exerts the most pronounced effect: the values range from −10.0 mV at pH 2 to −33.0 mV at pH 10. At pH 6, the zeta potential is approximately −5, −14, and −20 mV for the initial chalcopyrite, chalcopyrite with PAX, and the multicomponent xanthate, respectively. The more pronounced negative shift in the presence of multicomponent xanthate indicates a significant change in the interfacial electrical properties of the surface and is consistent with the adsorption of xanthate components onto the active sites of chalcopyrite. For galena, the initial surface was characterized by a positive ζ-potential in the acidic and slightly acidic ranges: approximately +24 mV at pH 2, +18 mV at pH 4, and +14 mV at pH 6. As the pH increased, the potential gradually decreased, approaching zero in the pH 8 region and reaching approximately −10 mV at pH 10. Following PAX treatment, a marked shift in the ζ-potential towards negative values was observed: from approximately +8 mV at pH 2 to −18 mV at pH 10. An even more significant change was observed following treatment with MX. Even at pH 2, the ζ-potential was around −2 mV, and as the pH increased further, it decreased to approximately −7 mV at pH 4, −15 mV at pH 6, −21 mV at pH 8, and −28 mV at pH 10. Thus, MX not only reduced the ζ-potential of galena but also shifted it into the negative region even at low pH values. The most pronounced difference between untreated galena and the galena-MX system was observed in the pH range 6–10. This change is consistent with the interaction of the xanthate components of MX with the surface sites of galena and the formation of a modified interfacial layer containing negatively charged or solution-facing functional groups.

However, the nature of the interaction, whether physical or chemical adsorption, cannot be unequivocally determined on the basis of ζ-potential data alone. For sphalerite, unlike galena, the initial surface exhibited a negative zeta potential across the entire pH range studied. Its value varied from approximately −8.5 mV at pH 2 to −29 mV at pH 10. PAX treatment led to a further shift in the potential into the negative region: approximately from −10.5 to −31 mV. In the presence of MX, this effect was enhanced, and the ζ-potential varied from approximately −13 mV at pH 2 to −35 mV at pH 10. Consequently, despite the initial negative surface charge of sphalerite, interaction with xanthate collectors was accompanied by a further increase in the negative electrokinetic potential, with the change being most pronounced for MX. The difference between PAX and MX persisted across virtually the entire pH range, indicating that single-component and multi-component xanthate collectors have different effects on the interfacial properties of sphalerite.

In this context, it is important to compare the electrokinetic results with the flotation data for sphalerite. Despite a more negative ζ-potential following MX treatment, the maximum sphalerite recovery in the experiments conducted was approximately 51.4%, which was significantly lower than that for chalcopyrite and galena. This indicates that the magnitude of the change in ζ-potential alone does not determine a mineral’s flotation activity. Flotation efficiency also depends on the nature and density of the adsorption layer, the degree of surface hydrophobization, and the specific interaction between the collector and the mineral’s surface centers.

A similar pattern was observed for pyrite; however, the absolute changes in ζ-potential were less pronounced than for galena. The initial pyrite was characterized by a positive ζ-potential of approximately +9 mV at pH 2, which decreased to approximately +2 mV at pH 4 and turned negative with a further increase in pH, reaching approximately −12 mV at pH 10. Following treatment with PAX, the ζ-potential of pyrite was negative across the entire range studied and varied from approximately −2.5 to −18 mV. MX caused a more pronounced negative shift: from approximately −5 mV at pH 2 to −21–22 mV at pH 10. Consequently, MX also interacts with the pyrite surface and alters the structure of its interfacial layer.

Differences in the sign of the zeta potential at low pH values may be due to variations in the composition of surface sites and the degree of oxidation of the minerals under investigation. In an acidic environment, surface groups undergo protonation. In particular, where hydroxyl groups are present on oxidized surface areas, their protonation may contribute to the formation of positively charged sites:

≡M–OH + H^+^ ⇌ ≡M–OH_2_^+^

where M denotes a surface metal atom. If the contribution from such centers predominates, the zeta potential may take on positive values.

However, acidification may also be accompanied by the dissolution of surface metal compounds and the formation of a sulfur-enriched surface layer. Therefore, for other minerals, protonation may reduce the negative value of the zeta potential without changing its sign. Thus, the observed differences reflect the combined influence of protonation, dissolution, and the composition of the surface layer.

The proposed explanation is considered a possible interpretation of the observed relationships: zeta potential measurements characterize the potential in the slip plane and, on their own, do not allow the composition of the charged surface centers to be unambiguously determined.

However, the electrokinetic interaction between MX and pyrite was not accompanied by a similarly high flotation recovery rate. The presence of an electrokinetic response confirms the interaction of the reagent with the interphase boundary; however, for effective flotation, a form and degree of surface modification is required that simultaneously ensures sufficient hydrophobization and stable attachment of the particle to the air bubble.

### 3.5. Adsorption Mechanism

**Figure 5:**
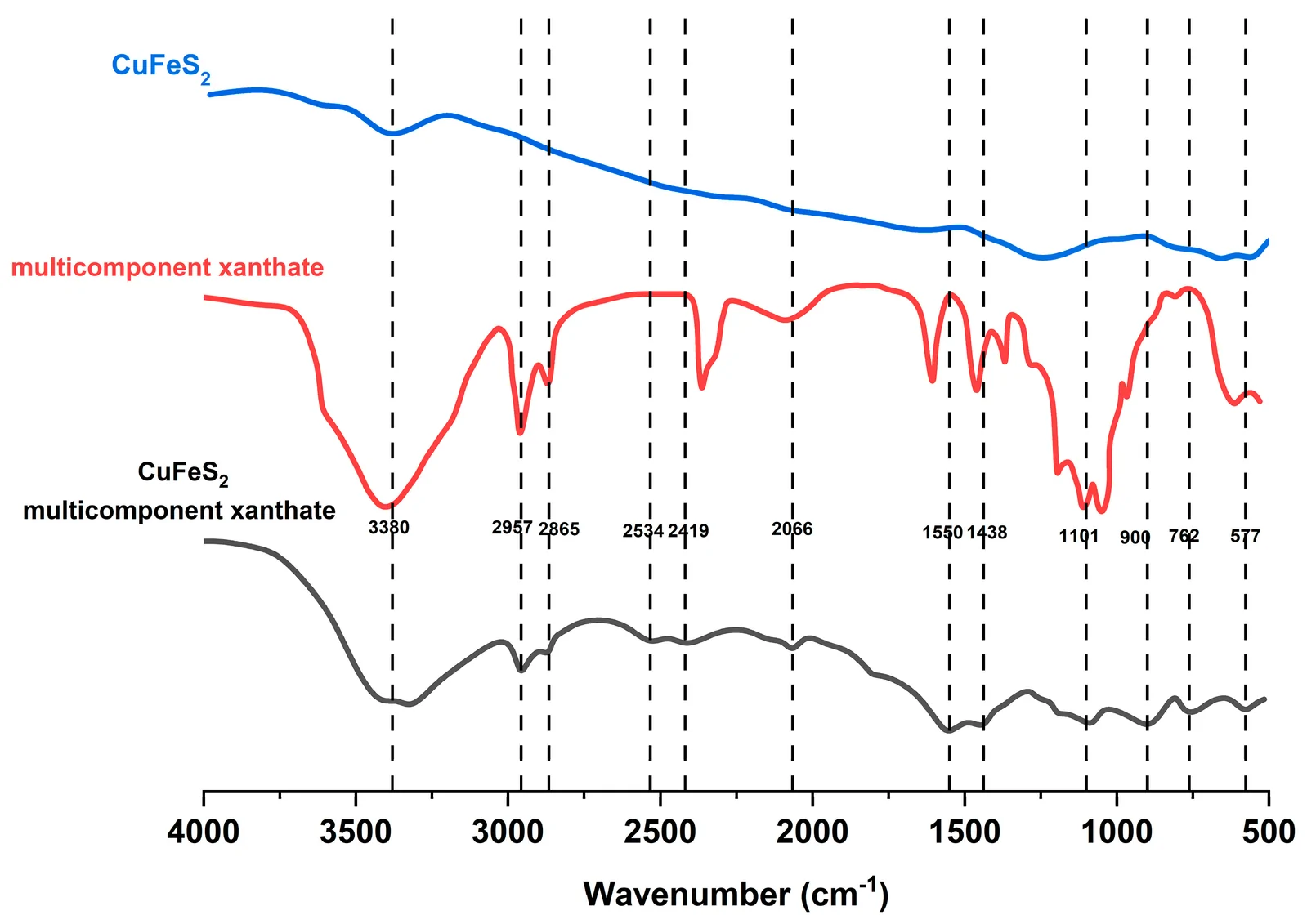
IR spectra of chalcopyrite, multicomponent xanthate, and chalcopyrite following treatment with multicomponent xanthate.

Figure 5 shows the IR spectra of the initial chalcopyrite (CuFeS_2_), the synthesized multicomponent xanthate, and the chalcopyrite following its interaction with the multicomponent xanthate. A comparison of the spectra allows us to assess changes in the functional composition of the mineral’s surface following reagent treatment and to establish the presence of adsorbed organic components.

The spectrum of the initial chalcopyrite is characterized by relatively low band intensities in the studied range, which corresponds to the predominantly inorganic nature of the mineral. The broad feature observed in the region around 3380 cm^−1^ may be associated with valence vibrations of O–H in adsorbed water and surface hydroxyl groups. The presence of oxygen-containing surface species on chalcopyrite is likely due to partial oxidation of its surface upon contact with aqueous and gaseous environments. In the low-frequency region of the spectrum, the changes may be associated with vibrations of the inorganic mineral matrix and surface products; however, it is difficult to unambiguously attribute these bands based solely on FTIR data.

Significantly more pronounced spectral features are observed for the synthesized multicomponent xanthate. The absorption bands at approximately 2957 and 2865 cm^−1^ correspond to valence vibrations of the C–H bonds in the alkyl groups. Their presence is consistent with the composition of the starting fusel oil, which contains a mixture of alcohols with various hydrocarbon radicals.

Following treatment with MX, the chalcopyrite spectrum exhibits weak features near the alkyl C–H stretching bands of the collector. However, the characteristic MX bands in the 1200–1000 cm^−1^ region are not sufficiently resolved to establish their shifts or unambiguously identify surface-bound xanthate species. Thus, the FTIR results may be consistent with the presence of organic components after treatment but do not independently demonstrate adsorption or chemisorption of MX.

**Figure 6:**
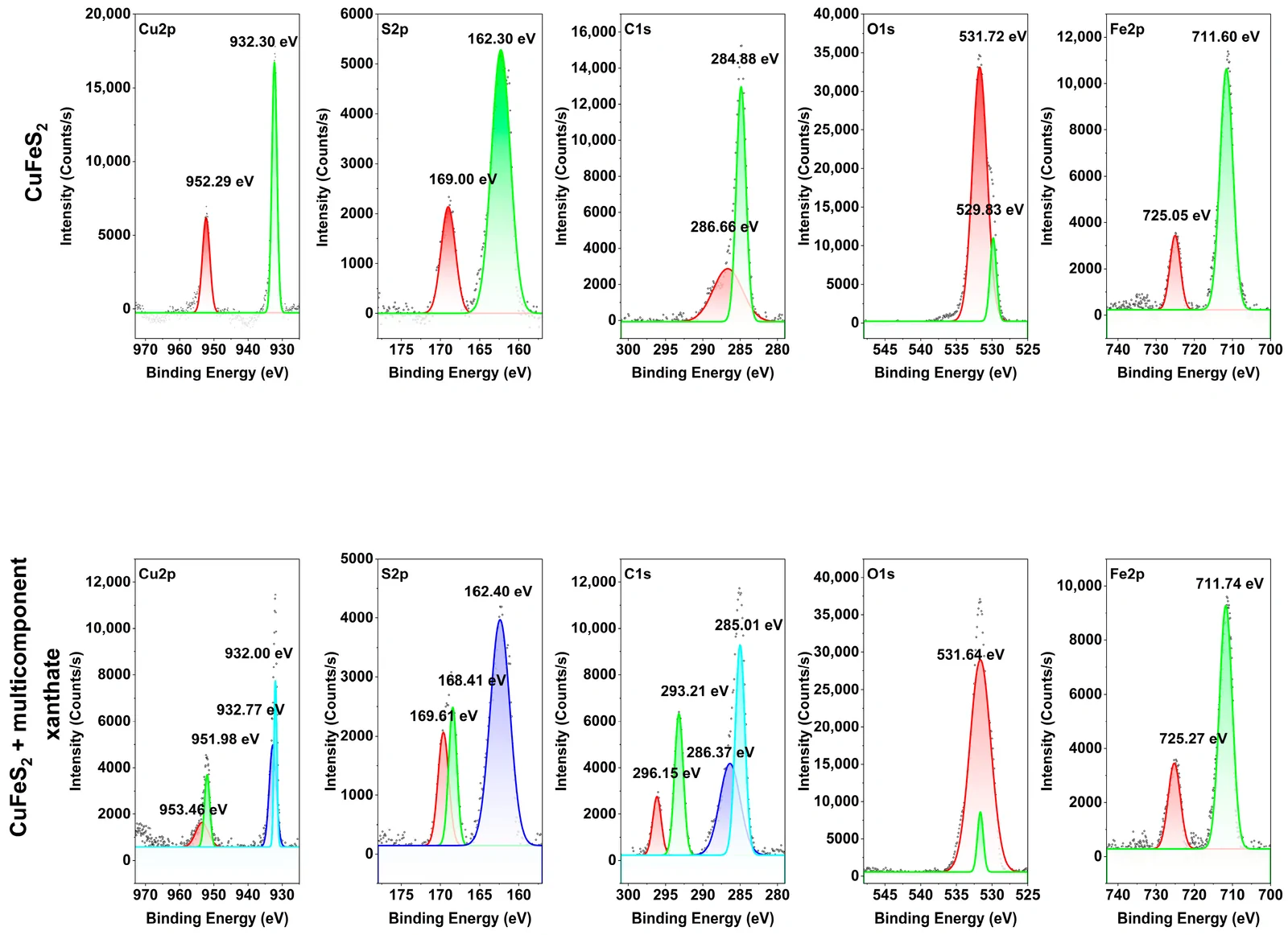
XPS spectra of Cu 2p, S 2p, C 1s, O 1s, and Fe 2p for the original chalcopyrite and chalcopyrite treated with a multicomponent xanthate.

To elucidate the mechanism of interaction between the multicomponent xanthate and chalcopyrite, XPS analysis of the surface was carried out before and after treatment with the collector (Figure 6). In the spectrum of the initial chalcopyrite, Cu 2p peaks are observed at 932.30 and 952.29 eV. Following treatment with the multicomponent xanthate, the main Cu 2p_3_/_2_ component is located at approximately 932.00 eV, whilst additional components appear at 931.98, 932.77, and 953.46 eV. The change in the shape and composition of the Cu 2p spectrum indicates a change in the chemical environment of the surface copper atoms following interaction with the reagent.

Changes are also observed in the S 2p region following treatment with the multicomponent xanthate. Untreated CuFeS_2_ exhibits spectral features at approximately 162.30 and 169.00 eV, whereas the treated sample shows features at approximately 162.40, 168.41, and 169.61 eV. The low-binding-energy region contains contributions from sulfur species associated with the mineral surface, which may overlap with signals from collector-derived sulfur. The high-binding-energy features are consistent with oxidized sulfur species and should not be assigned specifically to xanthate. In particular, the separation of 1.20 eV between the features at 168.41 and 169.61 eV is consistent with S 2p spin–orbit splitting; therefore, these features should not automatically be interpreted as two distinct chemical states. Overall, the observed spectral differences suggest surface modification following reagent treatment.

The C 1s and K 2p spectral region provides additional information on the surface composition of chalcopyrite before and after treatment with the multicomponent xanthate. Untreated chalcopyrite exhibits C 1s components at approximately 284.88 and 286.66 eV, tentatively attributed to adventitious hydrocarbon species and oxygen-containing carbon species, respectively. After treatment, the C 1s components are observed at approximately 285.01 and 286.37 eV. The additional peaks at 293.21 and 296.15 eV are assigned to the K 2p_3_/_2_ and K 2p_1_/_2_ spin–orbit doublet, respectively, rather than to carbon-containing species. The presence of potassium is consistent with the use of KOH in the synthesis of the collector. These potassium signals, however, do not independently establish adsorption of the xanthate anion or the formation of specific surface compounds. Accordingly, the changes in this spectral region should be considered together with the S 2p results, rather than treated as conclusive evidence of an adsorbed organic layer.

For the O 1s of the original chalcopyrite, components are observed at approximately 529.83 and 531.72 eV, whilst after treatment with the reagent, the main peak is located at approximately 531.64 eV. The changes in the O 1s spectrum may be attributed to both oxygen-containing surface products of chalcopyrite and oxygen from the xanthate group. In the Fe 2p spectrum, the main peaks of the initial and treated samples are located at approximately 711.60/725.05 and 711.74/725.27 eV, respectively. The slight shift in their positions, compared with the more pronounced redshift in the S 2p and C 1s peaks, suggests that the adsorption of the reagent primarily alters the chemical environment of the surface sulfur- and carbon-containing components, whilst the state of the iron is altered to a lesser extent.

### 3.6. Mechanism of Action of the Reagent

Unlike basic collectors, which contain normal hydrocarbon radicals (normal butyl and amyl xanthates), composite collectors exhibit greater surface activity due to the hydrophobic interaction between normal and isomerized hydrocarbon radicals. On the other hand, when these hydrocarbon radicals overlap, polar groups are exposed, which leads to an enhancement of the scavenging properties.

In [32], it is shown that, when mixtures of xanthates are used, their lower and higher homologues exhibit different abilities to oxidize on a mineral surface to form dixanthogenide. The longer the hydrocarbon chain of a xanthate, the more readily it oxidizes. This can be judged by the values of their standard electrode potentials [33].

In practice, various combinations of ethyl, isopropyl, and butyl xanthates are used. The proportion of each xanthate in a binary mixture is usually determined experimentally. Their total concentration, required for effective flotation of the target mineral, will increase as the potential deviates from the zero-charge value, for example, as the pH of the pulp increases. For example, for the flotation of galena at pH 8, a mixture of butyl xanthate and isopropyl xanthate is used; in this case, isopropyl xanthate, being readily adsorbed onto the surface of pyrite, reduces its floatability due to its short-chain radical. Butyl xanthate, on the other hand, readily floats galena at pH 8 [34]. The varying ratio of low- and high-molecular-weight xanthates in the mixture during the flotation process is due to the differing ratios of holes and electrons in the surface layer of the mineral being floated. The proportion of the stronger collector in the mixture increases with increasing electron concentration and decreases with increasing hole concentration [33].

## 4. Conclusions

This study investigates the flotation properties of a multicomponent xanthate collector (MX) derived from the alcohol components of fusel oil and the characteristics of its interaction with sulfide minerals. The multicomponent composition of the starting material accounts for the presence of various alkyl fragments in the resulting reagent, which is consistent with the results of GC-MS and NMR spectroscopy. Spectroscopic studies, taken together with data on the surface properties of the minerals, allow the action of MX to be linked to changes in the state and hydrophobicity of the sulfide surface.

Flotation studies revealed a marked dependence of MX efficiency on the nature of the mineral, the collector concentration, and particle size. The highest recovery was observed for chalcopyrite: for the −0.074 + 0.040 mm fraction at an MX concentration of 7.5 mg/L, it reached 81.9% after 3 min of flotation. For galena, the maximum final recovery was 73.9%; for sphalerite, 51.4%; whilst the recovery of pyrite did not exceed 23.9%.

## Figures and Tables

**Table 1:** Results of the chromatographic analysis of fusel oil.

Retention Time, min	Compounds	Probability of Identification, %	Percentage Content, %
1.53	Ethanol	98	29.7
2.08	1-Propanol	86	1.0
2.47	Isobutanol	95	4.6
3.91	Isoamyl alcohol	94	55.9
5.72	1-Pentanol	73	7.8
18.20	Phenylethyl Alcohol	67	1.0

**Table 2:** Theoretical yield of the composite xanthate.

Title	Chemical Formula	n, Moles	Molecular Mass, g/mol	Mass, g	Mass Fraction, %
Ethyl xanthate	C_2_H_5_OCS_2_K	0.271	160.30	43.44	39.61
Isobutyl xanthate	(CH_3_)_2_CHCH_2_OCS_2_K	0.026	188.35	4.90	4.47
Isoamyl xanthate	(CH_3_)_2_CH(CH_2_)_2_OCS_2_K	0.266	202.39	53.84	49.09
Amyl xanthate	CH_3_(CH_2_)_4_OCS_2_K	0.037	202.39	7.49	6.83

**Table 3:** Theoretical calculation of the hydrophilic-lipophilic balance (HLB) of individual xanthates and their composite mixture.

Component	Molecular Mass of the Hydrophilic Part of the Molecule, g/mol	Total Molecular Mass, g/mol	HLB of the Individual Xanthate	Mass Fraction in the Mixture, %	HLB by Mass	HLB Blends
Ethyl xanthate	92.00	160.30	11.48	39.61	4.55	10.07
Isobutyl xanthate	92.00	188.35	9.77	4.47	0.44	
Isoamyl xanthate	92.00	202.39	9.09	49.09	4.46	
Amyl xanthate	92.00	202.39	9.09	6.83	0.62	

## Data Availability

The original contributions presented in this study are included in the article. Further inquiries can be directed to the corresponding author.

## Appendix A

NMR Spectroscopic Characterization of a Multicomponent Xanthate

The structural features of the synthesized multicomponent xanthate collector (MX), derived from fusel oil, were investigated using ^1^H and ^13^C NMR spectroscopy. Spectra were recorded on a JNM-ECA 500 (JEOL) NMR spectrometer using D_2_O as the deuterated solvent.

In the ^1^H NMR spectrum of MX, a group of signals is observed in the δ 4.67–4.08 ppm range, assigned to the –OCH_2_– protons of the alkoxy fragments linked to the xanthate functional group. The presence of several closely spaced signals in this region (δ 4.666, 4.325, 4.286, 4.183, 4.109, and 4.075 ppm) is consistent with the presence of multiple alkyl fragments in the sample situated in different chemical environments.

Numerous resonances from methylene and methyl protons were detected in the aliphatic region of the spectrum. Signals at δ 1.92–1.61 ppm correspond primarily to β-methylene groups relative to the –OCH_2_– fragment, whereas the δ 1.49–1.13 ppm region characterizes the internal methylene groups of the hydrocarbon chains. An intense group of signals at δ 0.82–0.74 ppm is attributed to terminal methyl groups. Thus, the ^1^H NMR spectrum confirms the presence of alkyl fragments characteristic of the xanthate components of the synthesized collector.

The ^13^C NMR spectrum is particularly revealing regarding the multicomponent nature of the product. Unlike a pure amyl xanthate, for which a significantly smaller number of carbon resonances would be expected, the MX under study is characterized by a multitude of signals in the aliphatic and oxygen-containing regions. Specifically, a series of signals corresponding to carbon atoms bonded to oxygen is observed in the δ 73.30–81.77 ppm range, whereas numerous resonances in the δ 21.90–37.84 ppm range are attributed to methylene carbon atoms of various alkyl chains. The presence of several methyl signals in the δ 10.68–18.98 ppm range also indicates the heterogeneity of the product’s hydrocarbon fragments.

The set of these signals is consistent with the multicomponent nature of the synthesized product, which is to be expected for a collector derived from fusel oil—a substance containing a mixture of alcohol components. Consequently, the multiplicity of –OCHₓ, –CH_2_–, and –CH_3_ resonances should not be unequivocally interpreted as indicating the presence of impurities; rather, it may stem from the formation of a mixture of xanthate compounds with various alkyl substituents.
